# Immunological Effects of Human Milk Oligosaccharides

**DOI:** 10.3389/fped.2018.00190

**Published:** 2018-07-02

**Authors:** Vassilis Triantis, Lars Bode, R. J. Joost van Neerven

**Affiliations:** ^1^FrieslandCampina, Amersfoort, Netherlands; ^2^Department of Pediatrics, University of California, San Diego, San Diego, CA, United States; ^3^Wageningen University and Research, Cell Biology and Immunology, Wageningen, Netherlands

**Keywords:** human milk oligosaccharides, HMO, infection, immunity, infant, allergy, benefit, health

## Abstract

Human milk oligosaccharides (HMOs) comprise a group of structurally complex, unconjugated glycans that are highly abundant in human milk. HMOs are minimally digested in the gastrointestinal tract and reach the colon intact, where they shape the microbiota. A small fraction of HMOs is absorbed, reaches the systemic circulation, and is excreted in urine. HMOs can bind to cell surface receptors expressed on epithelial cells and cells of the immune system and thus modulate neonatal immunity in the infant gut, and possibly also sites throughout the body. In addition, they have been shown to act as soluble decoy receptors to block the attachment of various microbial pathogens to cells. This review summarizes the current knowledge of the effects HMOs can have on infections, allergies, auto-immune diseases and inflammation, and will focus on the role of HMOs in altering immune responses through binding to immune-related receptors.

## Introduction

**Figure 1:**
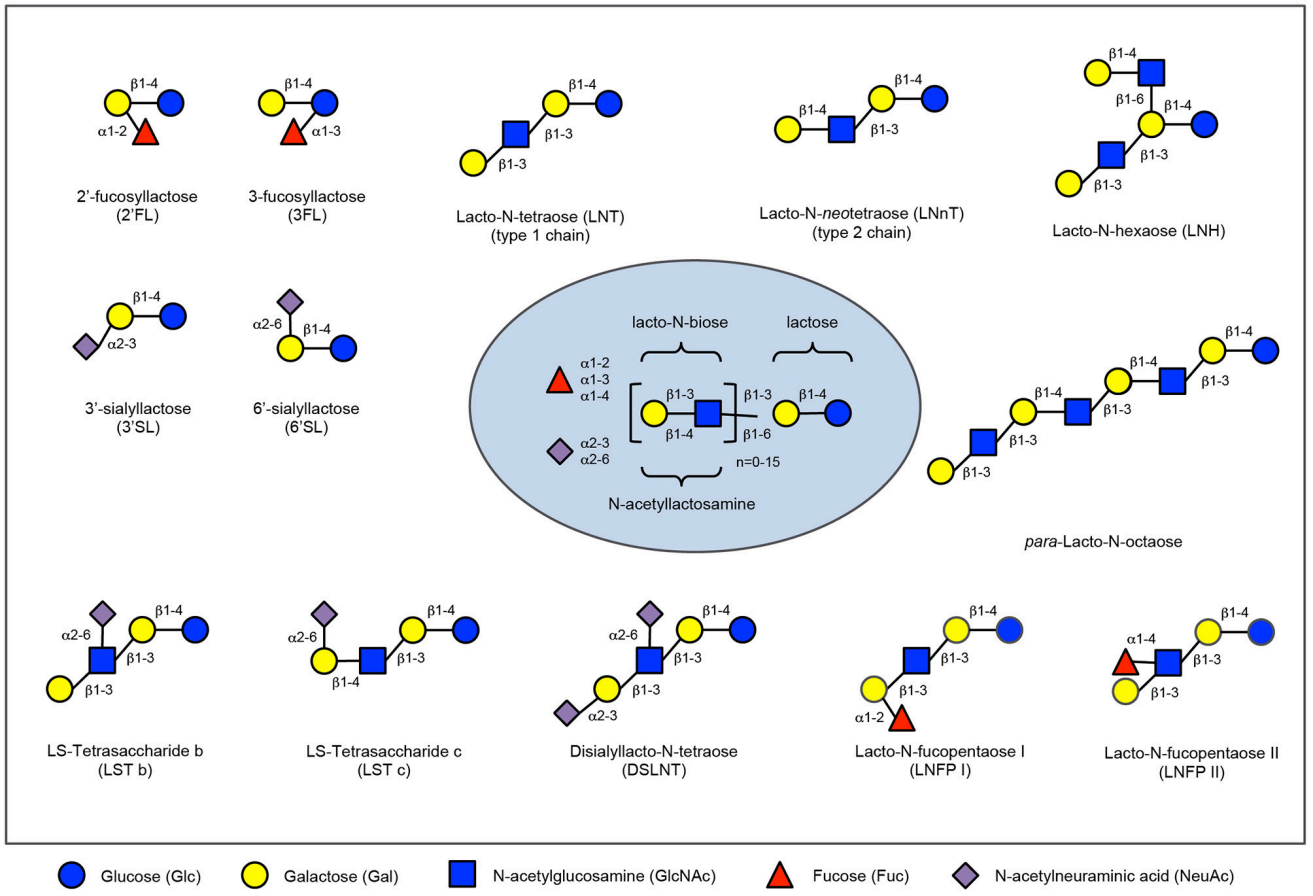
Human milk oligosaccharide composition blueprint. HMO composition follows a basic blueprint shown in the center. HMOs can contain 5 different monosaccharides in different number and linkages, namely glucose (blue circle), galactose (yellow circle), N-acetlylactosamine (blue square), fucose (red triangle), and sialic acid (purple diamond). All HMOs carry lactose at the reducing end. Lactose can be fucosylated or sialylated to generate the small HMOs 2′-fucosyllactose and 3-fucosyllactose or 3′-sialyllactose and 6′-sialyllactose, respectively (upper left corner). Alternatively, lactose can be elongated with type 1 or type 2 disaccharide units to form linear or branched HMOs (upper right corner). Elongated HMOs then can be sialylated (lower left corner) or fucosylated (lower right corner) or both sialylated and fucosylated (not shown). The HMOs in this figure are only a few relatively simple examples. So far, more than 150 different HMO structures have been identified.

Based on its richness in immune-related components like human milk oligosaccharides (HMOs), milk proteins and lipids, breastmilk can be seen as the first functional food humans encounter during their life (1). HMOs comprise a group of structurally complex, unconjugated glycans found in human breastmilk (see Figure 1). Although the amount and precise composition of HMOs varies depending on time of lactation and the genetic makeup of each woman as well as potential environmental exposures, human breast milk contains an average of 5–15 g of oligosaccharides per liter, making HMOs the third most abundant solid component of breast milk after lactose and lipids (2). Each oligosaccharide is built on a lactose backbone expanded by the addition of galactose, N-acetylglucosamine, fucose or sialic acid, branched and elongated in different ways, generating approximately 200 different structures identified to-date (3). As they are only minimally digested in the gastrointestinal tract, HMOs reach the colon intact or are absorbed in small quantities, reach the systemic circulation and are excreted in urine (4). In this way, they may exert a plethora of functions at multiple sites throughout the body and beyond the intestinal lumen and intestinal mucosal surfaces, including the urinary tract or the immune system. HMOs were first described as prebiotic substrates for the infant gut microbiota, promoting the establishment of bifidobacteria and lactobacilli, based on striking differences in microbiota composition between breastfed and bottle fed infants (5).

However, HMOs are now recognized to have various additional benefits for the developing neonate. HMOs may modulate neonatal immunity by altering host epithelial and immune cell responses in the infant gut (6), modify immune responses systemically or act as soluble decoy receptors to block the attachment of various microbial pathogens to cell surface receptors (7), not only in the intestine but also in other sites such as the urinary tract (8). The benefits of HMOs can extend to health outcomes beyond infancy such as allergies (9) or cognitive functions (10), making HMOs the focus of intense current scientific research with increasing number of studies unraveling their role in human physiology.

This review summarizes recent findings, discusses the proposed modes of action, and identifies future prospects and scientific challenges, with a focus on immunity and infection.

## HMO absorption

HMOs are resistant to digestion in the infant GI tract (11). Both neutral and acidic HMOs can cross the epithelial barrier, but active transport over intestinal epithelial monolayers has only been demonstrated for neutral HMOs (12). These findings suggest that HMOs may be taken up into the human body. Indeed, HMOs have been detected in feces and urine of breastfed infants (13–17), but also directly in the peripheral blood (18–21). However, lower concentrations of HMOs are detected in blood compared to urine, which may be a reflection of accumulation in urine from a larger volume of blood. For example, concentrations of 2′-fucosyllactose (2′FL), were around 1.5 mg/l in peripheral blood and 100 mg/l in urine (20).

Absorption of orally administrated single HMOs was also shown in an adult rat model showing indeed that the intestinal epithelium is permeable to HMOs although to a different extent in infancy and adulthood (22). Therefore, these publications indicate that HMOs may, in addition to effects in the GI tract, have effects throughout the human body. Such effects can be conveyed directly through binding to receptors for HMOs, or indirectly via induction of short chain fatty acids and other metabolites produced by the microbiota.

## Potential HMO receptors, their expression and function

### Potential HMO receptors

Several classes of lectins (glycan-binding proteins) have been described in the literature that have different functions and ligand specificities, namely galectins, siglecs, c- type lectins, and selectins. Different HMOs can bind to these different types of receptors on human cells, primarily expressed on cells of the immune system.

Galectins are lectins that bind N-acetyllactosamine or lactose containing sugars (23–25). Galectins can also bind sulfated, sialylated or fucosylated galactose moieties (25). The work of Hirabayashi et al elegantly shows the oligosaccharide specificity of galectins for several HMO structures (23, 25, 26). More recently, Prudden et al. confirmed binding of HMOs with a terminal type 1 and 2 LacNAc to galectin 9 with a preference for type 1 structures on a solid surface (27). Similar findings were reported for HMO binding specificity for galectins in solution, corroborating these initial studies (28, 29).

Another family of lectins involved in HMO binding are the sialic acid binding immunoglobulin- like lectins (Siglecs). Siglecs have been shown to bind sialylated HMOs (30). Sialyllactose has been shown to bind to sialoadhesin (Siglec-1) (31), but also to Siglec-5 and Siglec-10 (32), Siglec-7 (33), and Siglec-9 (34). However, the affinity of sialyllactoses for Siglecs are relatively low.

In addition to galectins and siglecs, HMOs also interfere with another family of lectins involved in cell adhesion, the selectins (2, 35). Selectins bind to glycans that carry sialylated Le bloodgroup epitopes (36), which are sialylated and fucosylated lacto-N-bioses (Galβ1-3GlcNAc) or N-acetyllactosamines (Galβ1-4GlcNAc)—very similar to HMOs. In fact, HMOs contain Le blood group antigens (37) and are able to reduce selectin-mediated cell–cell interactions (38, 39). In addition, HMOs have been shown to interact with selectins (40), and integrins (39).

Finally, HMOs can bind to C-type lectins like DC-SIGN and Dectin-1. C-type lectins containing an EPN-motif (Glu-Pro-Asn) have high specificity for mannose- and fucose terminating glycans, whereas the presence of a QPD-motif (GlnPro-Asp) is important for galactose-or N-acetylgalactosamine(GalNAc) terminating glycans (41). HMOs were shown to bind specifically to DC-SIGN expressed by DCs (42). Although HMO binding to DC-SIGN seems to be weaker than binding to galectins, it was shown that structures containing α-linked fucose could bind to DC-SIGN (34). The results were also confirmed by binding of DC-SIGN to beads derivatized with 2′-FL or 3-FL, but not with LNT.

A limited number of reports have also discussed the possibility of binding of HMOs to other receptors belonging to the Toll like receptor (TLR) family that typically bind to pathogen-related molecules. TLR-4 dependent effects of HMOs have been described in two papers in which HMOs tested *in vivo* required the expression of TLR-4 for their effect (43, 44). However, formal demonstration of the binding of the HMOs (3′SL and LNFPIII) to TLR-4 in direct binding assays was not provided. In addition, in relation to TLR-signaling of HMOs, a recent paper highlighted the effect that low level LPS contamination of the commercially available HMO 3′SL can have in these studies, indicating that caution is warranted when studying TLR-mediated effects (45).

An overview of putative receptors for HMO is shown in Table 1.

**Table 1:** Putative receptors for HMOs on the immune system.

**Type of lectin**	**Name**	**Function of receptor**	**Ligand specificity**	**Known HMO ligands**	**Expression**	**Reference**
C-type lectins	DC-SIGN	Immune (Antigen presentation)	α-fucosylated structures, mannose	2′-FL, 3FL, LNFP-III, LNFP-IV, LNDFH-I	Antigen presenting cells	(34)
Siglecs	Siglec-5, Siglec-9	Immune	sialylated HMO, α2,3- and α2,6-linked sialic acids	3′ and 6′ Sialyllactose	Neutrophils, monocytes, dendritic cells	(34)
Galectins	Galectins 1, 2, 3, 7, 8. 9	Immune	N-acetyllactosamine or lactose	LNnT LNT NFP-I LNFP-II LNFP-III LNDFH FucLac a-GalLac but not 6SL	Intestinal cells, lymphocytes, antigen presenting cells	(23, 25, 26)
Selectins		Leucocyte adhesion	Sialylated and fucosylated lacto-N-bioses (Galβ1-3GlcNAc) or N-acetyllactosamines	sialyl-Lewis x	Leucocytes, endothelium	(37, 39)

### Expression profiles and functions of potential HMO receptors

Galectins are mainly expressed on T cells, and can regulate T cell function (46), but are also present on intestinal epithelial cells (47–49), and on antigen presenting cells and granulocytes (25). Galectins can convert signals into the cell after binding to their ligands directly, but galectins can also be secreted, after which they bind to glycoproteins or receptors at cell surfaces and hence can regulate cell functions (50–52). Binding of HMOs or lactose can thus have direct effects or inhibit the interaction of galectins with their ligands on other cells.

Siglecs are involved in the immune system in multiple ways (53). Siglecs 1–16 are expressed on a variety of blood cells, including monocytes, macrophages, dendritic cells, neutrophils, eosinophils, basophils, and NK cells (53, 54). In contrast to galectins and Dectin-1, Siglecs are not expressed by intestinal epithelial cells. Many of the Siglecs have an intracellular immunoreceptor tyrosine-based inhibitory motif (ITIM), and are thus known as regulators of immune responses.

Selectins are cell adhesion molecules that mediate the earliest stages of leukocyte trafficking. At sites of inflammation, leukocytes need to migrate from the blood stream through the endothelium into sub endothelial regions of inflammation (55, 56). Induced by pro-inflammatory cytokines, endothelial cells express P- and E-selectin, which bind to glyco-conjugates on leukocytes passing by with the blood stream. This initial contact decelerates the leukocytes and makes them roll over the endothelial cell layer. Subsequently, additional adhesion molecules bring leukocytes to a complete stop and facilitate their transmigration into sub endothelial regions. Initial selectin-mediated rolling is essential for leukocyte extravasation and mucosal infiltration.

Sialylated HMOs have been shown to interact with selectins (40), and integrins (39), and affect leukocyte-endothelial cell and leukocyte-platelet interactions (39, 57–59). Similarly, sialylated HMOs reduce PNC formation and subsequent neutrophil activation in an *ex vivo* model with whole human blood (38). In both cases, non-sialylated HMOs are ineffective and pooled HMOs are more effective than monovalent sialyl-Le X, indicating the importance of Sia and suggesting potential multivalent interactions with higher molecular HMOs that carry more than one sialylated blood group epitope.

C-type lectins are primarily expressed by antigen presenting cells (monocytes, macrophages, dendritic cells) and are of crucial importance for regulating immune responses to pathogens. The can be divided into four subgroups, the sialo-glycoprotein receptor family (e.g., DC-SIGN), the dectin-1 subfamily of asialo glycoprotein receptors (e.g., Dectin-1), the DCIR subfamily (e.g., DCIR), and the Mannose receptor family (e.g., CD206) for a review see Geijtenbeek and Gringhuis (60).

In general, c- type lectins are primarily expressed on dendritic cells and macrophages, and play a role in the internalization of saccharide-containing antigens, resulting in antigen presentation (41). However, dectin-1 can also be detected on intestinal epithelial and on M cells, and play a role in IgA transcytosis (61–63). Apart from promoting antigen presentation, some c- type lectins like DCIR may—just like Siglecs—contain an immunoreceptor tyrosine-based inhibitory motif (ITIM) -motif in their intracellular domains, that inhibit immune activation.

DC-SIGN interacts with a variety of pathogens, including HIV-1, and binding of HMOs inhibited the transfer of HIV-1 to CD4+ T lymphocytes. These data may suggest that oligosaccharides act systemically and are thereby modulating the immune response in a microbiota-independent manner. In addition, recent publications have also demonstrated that the c-type lectin Dectin-1 can modulate innate immune function, possibly explaining the cross-protection against other pathogens seen after vaccination (64, 65).

The functions of these receptors thus indicates that binding of HMOs to these structures may result in regulation of adaptive and innate immune protection against infection and inflammation.

## Effects of HMOs on infection, allergy and immune parameters in human studies

As can be seen in Table 2, there are currently only a few infant studies on effects of HMOs on infection and immune function. Most of these studies are observational studies on breastfeeding infants, correlating HMOs in breastmilk with these outcomes. Placebo controlled studies with HMOs have been performed but have to date focused on safety rather than on anti-infective and immunomodulatory effects (18, 77).

**Table 2:** Human studies with HMOs and measured outcomes.

**Title of study**	**Health-related effects**	**HMO used**	**Target group**	**Study setup**	**Outcome of effect HMO (Short)**	**Reference**
Fucosyltransferase 2 non-secretor and low secretor status predicts severe outcomes in premature infants.	Mortality, necrotizing enterocolitis (NEC), sepsis	Breastmilk	Infants (*n* = 410)	Observational study	Mortality, NEC and gram - sepsis increased in infants receiving low secretoe status breast milk	(66)
Human milk oligosaccharides are associated with protection against diarrhea in breast-fed infants.	Diarrhea	Breastmilk	Infants (*n* = 93)	Observational study	(1) High levels of 2-FL in breastmilk protective against Campylobacter diarrhea (2) High levels of lacto-N-difucohexaose (LDFH-I), also a 2-linked fucosyloligaosaccharide, protective against calicivirus diarrhea	(67)
Innate protection conferred by fucosylated oligosaccharides of human milk against diarrhea in breastfed infants	Diarrhea	Breastmilk	Infants (*n* = 93)	Observational study	Breast milk with higher 2-linked to non-2-linked fucosyloligosaccharide ratios affords greater protection against infant diarrhea	(68)
Early consumption of human milk oligosaccharides is inversely related to subsequent risk of respiratory and enteric disease in infants.	Diarrhea and respiratory infection	Breastmilk	Infants (*n* = 49)	Observational pilot study	LNF-II levels in breast milk and in infant feces at 2 weeks of age (as representative of total HMO) associated with fewer infant respiratory problems and gastropintestinal problems by week 6 and week 12	(69)
FUT2-dependent breast milk oligosaccharides and allergy at 2 and 5 years of age in infants with high hereditary allergy risk	Eczema	Breastmilk	Infants at risk for allergy (*n* = 266)	Observational study (in placebo arm of controlled study)	At 2 years, but not at 5 years, FUT2-dependent oligosaccharides associated with lower IgE-associated eczema manifestations. Only in C-section-born infants with high allergy risk	(70)
Human milk oligosaccharides and development of cow's milk allergy in infants	CMA	Breastmilk	Infants with (*n* = 35) and children without CMA (*n* = 39)	Observational study	Infants receiving breast milk with low LNFP III levels more likely to become affected with CMA than infants receiving higher levels of LNFP III	(9)
Effects of infant formula with human milk oligosaccharides on growth and morbidity: A randomized multicenter trial	Respiratory infection (bronccitis) and antibiotic use	Formula containing 2′fucosyllactose (2′FL) + lacto-N-neotetraose (LNnT)	Infants receiving cow's milk-based infant formula (*n* = 87) vs. the same formula with 2′FL and LNnT (*n* = 88)	Multicenter, randomized, double-blind trial	Infant formula supplemented with 2′FL and LNnT associated with lower parent-reported morbidity (particularly bronchitis) and medication use (antipyretics and antibiotics)	(71)
Infants fed a lower calorie formula with 2′-fucosyllactose (2′FL) Show Growth and 2′FL Uptake Like Breast-Fed Infants	Growth	Formula supplemented with 2-Fucosyllactose (2′FL) and galactooligosaccharides (GOS)	Infants exclusively formula-fed in 3 groups: (1; *n* = 101 control formula GOS 2; *n* = 104 formula high GOS and low 2′FL 3; *n* = 109 medium GOS and medium 2′FL) or breastfed (*n* = 106) from enrollment to 4 mo of age	A prospective, randomized, controlled, multicenter growth and tolerance study	Growth and 2′FL uptake similar to breast milk	(18)
Similar to those who are breastfed, infants fed a formula containing 2′-fucosyllactose have lower inflammatory cytokines in a randomized controlled trial	Immune parameters	Formula supplemented with 2-FL and GOS	Infants exclusively formula-fed in 3 groups: (1; *n* = 75 control formula GOS 2; *n* = 76 formula high GOS and low 2′FL 3; *n* = 78 medium GOS and medium 2′FL) or breastfed (*n* = 86) from enrollment to 4 mo of age	Observational substudy nested within a randomized, double-blind, controlled study	Infants fed formula supplemented with 2′-FL exhibit lower plasma and *ex vivo* inflammatory cytokine profiles, similar to those of a breastfed reference group	(72)
Human milk oligosaccharide concentration and risk of postnatal transmission of HIV through breastfeeding.	HIV transmission	Breastmilk	Breast milk of HIV-infected women who did not transmit HIV despite breastfeeding (*n* = 86), and uninfected women (*n* = 36)	Nested case-control study was conducted within a larger cohort study	(1) Higher concentrations of non-3′-SL HMOs were associated with protection against postnatal HIV transmission (2) A trend toward higher concentrations of lacto-N-neotetraose (LNnT) being associated with reduced transmission	(73)
Human milk oligosaccharides differ between hiv-infected and hiv-uninfected mothers and are related to necrotizing enterocolitis incidence in their preterm very-low-birth-weight infants	NEC, HIV infection	Breastmilk (secretor/nonsecretor)	HIV infected mothers (*n* = 41 of which 22 secretor, 19 non-secretor) and non-infected mothers (*n* = 41 of which 20 secretor, 21 non-secretor)	Substudy of a larger clinical trial on HIV-infected and HIV-uninfected mothers and their preterm infants	(1) HIV-infected mothers have higher relative abundances of 3′-SL in breastmilk (2) Low concentrations of DSLNT in brestmilk increased infant's risk of NEC	(74)
Growth and Morbidity of Gambian Infants are Influenced by Maternal Milk Oligosaccharides and Infant Gut Microbiota.	Morbidity	Breastmilk	Mother/infant pairs (*n* = 33, of which 21 secretors and 22 non-secretors)	Sub-study embedded within a randomized trial	(1) Higher breast milk levels of lacto-N-fucopentaose I (secretor) associated with decreased infant morbidity (2) Higher breast milk levels of LNT (non-secretor) associated with higher infant morbidity 3) Breast milk levels of 3′-sialyllactose indicator of infant weight-for-age	(5)
Oligosaccharide composition of breast milk influences survival of uninfected children born to hiv-infected mothers in Lusaka, Zambia	HIV infection, mortality	Breastmilk	HIV-infected children (*n* = 103) and HIV exposed uninfected children (*n* = 143).	Nested case-cohort study	High levels of fucosylated HMOs in breastmilk of mothers of HEU children protective against mortality	(75)
The impact of breastfeeding on nasopharyngeal microbial communities in infants.	Respiratory infection	Breastmilk	Infants receiving exclusive breastfeeding (*n* = 101) vs. and exclusive formula feeding (*n* = 101)	Case-cohort analysis	(1) Association between breastfeeding and microbial community composition in the upper respiratory tract (2) Possible link to protective effect of breastfeeding on respiratory infections and wheezing in early infancy	(76)

Four of these studies showed an effect of HMOs on prevention of diarrhea (67, 69), respiratory tract infections (69), and severe outcomes like sepsis and death (75). Morrow et al. showed in another study that HIV exposed, non-infected children receiving breastmilk of secretor+ mothers have a reduced risk of early mortality compared to secretor—breastfeeding (66).

Also, in relation to cow's milk allergy, the level of Lacto-N-fucopentaose (LNFP) III in breast milk correlated with the prevalence of cow's milk allergy (9). Similarly, Sprenger et al. reported that FUT2-dependent breast milk oligosaccharides, with the levels of 2′FL as proxy for secretor status, were associated with lower levels of IgE-mediated allergies and eczema (70).

Finally, Biesbroek et al. reported recently that 6 week old breastfed children have a different nasopharyngeal microbiota, suggesting that milk components like HMOs may influence the nasopharyngeal microbiota composition—which may contribute to the protective effect of breastfeeding on decreased respiratory infections (76).

It should be stressed that none of these studies have formally demonstrated direct effects of HMOs, and that other breastfeeding components may be associated with the effects described. Only in a recent study (71) the administration of 2′-FL in combination with LNnT could reversely correlate with parentally reported episodes of bronchitis, lower respiratory tract infections, and use of antipyretics or antibiotics at different ages.

However, as reviewed in detail in section Effects of HMOs on Infection, Allergy and Immune Parameters in Human Studies and in several recent reviews (2, 35, 78–88), quite some information is available of effects of effects of HMOs on microbiota composition, pathogens, and pathogen adhesion *in vitro*, as well as on infection *in vivo*. In addition, effects on intestinal epithelium and barrier function, as well as immune function have been described in these reviews and the underlying literature.

In infants another study showed that the secretor or non-secretor genotype of mothers of infants that were breastfed correlated with enterocolitis (low secretor) and sepsis (non-secretor) (66).

It has been shown as well that the amount of 2-linked fucosylated oligosaccharides in breast milk inversely correlates with the incidence of diarrhea in infants (89), and similarly the amount of fucosyl oligosaccharides in breast milk inversely correlates with the severity of infection with E coli that has a stable (68). Similarly, the amount of 2FL inversely correlated with Campylobacter diarrhea (89).

Bode et al demonstrated that the risk of HIV transmission in breastfeeding children of HIV infected mothers inversely correlates with HMO concentration (73). In another study the amount of LDFH-1 in breast milk inversely correlated with norovirus diarrhea (67). In addition to effects of HMOs on intestinal infections, HMOs have been linked to other infections such as infections of the urogenital tract (8, 90), and airway infection (69, 91, 92).

Such results were expanded lately with measuring inflammatory cytokines in systemic circulation if infants receiving infant formula supplemented with 2′-FL (72).

Much more is known about the direct effects of HMOs on pathogens, on adhesion and infection in *in vitro* models, and in animal models.

## Effects of HMOs on immune function and infection in *in vitro* and animal studies

### Effects on bacterial adhesion and infection

HMOs have been shown to prevent adhesion of several potential pathogens to epithelial surfaces in the intestine and other organs by acting as decoy receptors for bacterial pathogens like Campylobacter or *E. coli* (86, 93–95).

Several recent manuscripts report specific effects of isolated HMOs. For example, Weichert et al. showed that 2′FL, and to a lesser extent 3FL, reduce the adhesion of Campylobacter, EPEC, Salmonella and Pseudomonas, although the inhibitory effects were very small (96). Sialylated oligosaccharides were shown to reduce adhesion of EPEC (97, 98). In addition to reducing the adhesion of entire bacteria, HMOs may also compete with binding of bacterial toxins and mitigate their diarrheal activity (99, 100).

However, not always is the beneficial effect of HMOs to bacterial infection arising from preventing association or invasion of the pathogen. For example, HMOs can alter gene expression in intestinal cells that can block infection of Listeria monocytogenes (101), or have a direct effect on the growth of pathogens as was shown for neutral HMOs and especially LNT and LNFP I against group B Streptococcus (102). Similarly, HMOs were shown to modulate hyphal induction in Candida albicans, which is necessary for invasion of the intestinal epithelium (103). Another mechanism could be attenuation of pathogenic virulence through metabolites from fermentation of HMOs from the intestinal microflora. That seems to be at least partially the case for *Escherichia coli* O157:H7 and Salmonella typhimurium (104). When bifidobacteria of human and bovine origin were grown on medium containing 3′-SL, they could produce metabolites that could block expression of virulence genes in both pathogens. In addition, HMOs may have an indirect effect on bacterial infection by reducing epithelial inflammatory responses as it has been shown for 2′-FL and Campylobacter-induced inflammation (105).

While 2′-FL protected against adherent-invasive *E. coli*-induced pathology in mice (106), it failed to improve *E. coli*-induced diarrhea in piglets (107). These contradicting results could be explained by the difference in model, virulence of different *E. coli* strains, dosage and timing of administration of pathogens and HMOs, etc.

### Effects on intestinal viruses

Shang et al. demonstrated that different HMOs can bind to norovirus (LNFPII and 2′FL) and Norwalk virus (LNFP I and LNDFHI), indicating that several potential Noro- and Norwalk virus-binding glycans are present in HMOs that can play a role in viral infection (108). Notably, they also showed that LNFP III-HSA and 2′-FL-BSA – but not heir monovalent forms (LNFP III-Gly and 2′-FL-Gly) bound to VA287 capsids. This suggests that polyvalent oligosaccharides on a carrier protein may be more potent in anti-adhesion effects than their monovalent sugars themselves. However, recently it was also shown that 2′-FL can block both the GI.1 and GII.17 noroviruses from binding to HBGAs (109).

In addition to effects on gut bacteria, HMOs can also have effects on viral pathogens as rotavirus, norovirus, and HIV [reviewed in (85)].

In Rotavirus infected piglets, HMO-supplemented piglets had a shorter duration of diarrhea compared to the control Group (110, 111). There have been several HMO structures identified that bind the glycan rotaviral receptor VP8^*^. The sialic acid containing HMOs inhibited rotavirus infection *in vitro*, but *in vivo* both neutral HMOs and sialic acid containing HMOs decreased replication during acute RV infection *in situ*. These data are confirmed by recent *in vitro* findings where 2′-FL, 3′-SL, and 6′-SL could block infectivity of human rotaviral strains in cells (7). Apparently simple HMO structures can act as decoy receptors for viruses. However, since there are differences in the infectious mechanism of porcine and human rotaviral strains extrapolation from porcine to human models can be treacherous and more research is needed to clarify the role of HMOs in rotaviral infections.

### Effects on respiratory viruses

In addition to effects on intestinal pathogens, HMOs have been suggested to also play a role in infections from respiratory viruses. For example, 2′FL was shown to decrease RSV viral load, whereas LNnT and 6′SL decreased influenza viral load. Also effects were observed on innate cytokines in response to both viruses (92) suggesting an effect of HMOs on respiratory virus infection. This is supported by an early study by Stepans-Flanders on the fact that HMO consumption is inversely linked to respiratory infection (69). In this study higher LNFPII levels in breastmilk correlated with decreased respiratory and gastrointestinal infections in early infancy.

Immobilized 3′SL and 6′SL haven been shown to prevent infectivity of influenza viruses as a result of blocking the haemagglutins of influenza viruses (112, 113), and Yu et al. identified a number of additional sialic acid containing HMOs that bind to influenza virus (114). The effects of these HMOs was confirmed in a functional infection assay *in vitro*, where 6′SL and LNnT were shown to reduce the viral load of influenza in airway epithelial cells, and 2′FL did the same for respiratory syncytial virus (RSV) (92). In one recent *in vivo* study 2′FL enhanced responses to vaccination in mice (115). The mechanism was postulated to involve also a direct effect of 2′FL on dendritic cells as shown *in vitro*. However, concentrations used in their experiments were more than 1000-fold higher than what has been described to be found in circulation (20), warranting further clarification and research on the mechanism of action and relevance to human breast-fed infants.

### Enterocolitis

In relation to necrotizing enterocolitis, Jantscher-Krenn et al. noted in a rat model that disialylated LNT (DSLNT) increased survival rates and improved pathology scores (116), while low amounts of DSLNT in mother's milk could be a predicting risk factor for the development of NEC in premature infants (117) corroborating the previous findings. More HMOs could have a beneficial effect in NEC as it was shown also in a rat study where 2′-FL ameliorated the pathology of NEC, however there was no association between 2′FL and NEC risk in the corresponding human cohort (115). Similar observations were made for rats fed sialylated galacto-oligosaccharides (Sia-GOS) (118). Studies in mice have also shown a beneficial effect of 2′-FL in an induced NEC model (119). However such effect could not be seen in a piglet model where piglets born with caesarian section were fed control formula or formula supplemented with 2′-FL and were let to develop NEC spontaneously (120). Differences between induction vs. natural progression to NEC or species differences could account for such outcomes. In infants another study showed that the secretor or non-secretor genotype of mothers of infants that were breastfed correlated with enterocolitis (low secretor) and sepsis (non-secretor) (66).

### Effects of HMOs on intestinal epithelium

In another study immunomodulation by 2′FL *in vivo* was shown to be dependent on the downregulation of CD14 on intestinal epithelial cells (106). As CD14 is a co-receptor for LPS and is involved in TLR-4 signaling, this may lead to decreased inflammatory responses in the intestine after exposure to LPS.

In a recent study by He et al. the effect of colostrum oligosaccharides on gene expression in fetal immature intestinal mucosa was tested (121). They identified several immune related pathways were induced by HMOs, such as Immune cell communication, homeostasis, and intestinal immune differentiation. HMOs could reduce the response to TLR stimuli, and induced cytokines that are involved in tissue repair. 3′, 4', and 6′ galatosyllactoses were the most potent oligosaccharides.

Sialyllactose has been described to be able to promote the differentiation and growth of human intestinal epithelial cells as measured by upregulation of expression of alkaline phosphatase (122). Alkaline phosphatase is a molecule that is important in maintaining gut barrier function, possibly through the inactivation of LPS, by cleaving of a phosphate group from LPS. This suggests that on the one hand epithelial cells may yet have a receptor that recognizes Sialyllactose, and that Sialyllactose may be beneficial for promoting a good epithelial barrier in the gut.

In addition, two recent papers suggest that SL or goat milk oligosaccharides containing SL may have an effect on epithelial cells via activating through TLR4 (43, 123).

In contrast, another paper showed that 3′SL had anti-inflammatory activity by reducing the expression of IL-12 and IL-8 in Caco-2 cells, mediated via NFkB, and stimulates the anti-inflammatory nuclear receptor PPARg (124). Especially neutral HMOs have been shown to have an anti-inflammatory effects on the intestinal epithelium in *in vitro* inflammatory models (106, 121).

Lane compared effects of HMOs and BMOs on gene expression in HT-29 cells, noting that “both treatments including a response to stimulus, signaling, locomotion, and multicellular, developmental and immune system processes” (125).

Combined, these studies suggests that milk oligosaccharides contribute to the development and maturation of the intestinal immune response.

### Effects of HMOs on immune function

Acidic HMOs (but not acidic cow's milk oligosaccharides) were shown to induce IFN-g and IL-10 in human cord blood T cells, and could decrease IL-4 production in allergen-specific T cells (126). These data suggest that acidic HMOs may downregulate Th2 responses in infants as well.

Such results were expanded lately with measuring inflammatory cytokines in systemic circulation if infants receiving infant formula supplemented with 2′-FL (72). In this study, supplementation of 2′-FL alone could lower levels of TNFα, IL-1α, IL-1β, and IL-6 resembling those found in breast fed infants. In early studies LNFP III and LNnT were shown to have immunosuppressive effects (127, 128), and LNFPIII can induce IL-10 in macrophages (129, 130). Unexpectedly, a link with helminth infections exists. It is now known that upon infection with helminths confers a protective effect on allergy development (131, 132). Schistosoma eggs, but not the helminth itself, induce potent IL-10 responses that inhibit Th2 responses (133, 134). These effects are at least in part mediated by the oligosaccharides LNFPIII GalNAcβ1-4(Fucα1-2Fucα1-3) GlcNAc (LDN-DF) and Lewis-X that is present in the egg shells. These oligosaccharides are also found in breast milk, suggesting a functional anti-allergic/anti-inflammatory role of these HMOs. Likewise, Comstock et al. demonstrated a similar effect of HMOs *in vivo* in piglets, where HMOs induced IL-10 levels and inhibited T cell proliferation (135). The same was noted by Hester et al that showed enhanced T helper type 1 (interferon-gamma) and anti-inflammatory (interleukin-10) cytokines in the ileum in response to HMO supplementation of piglets in a rotavirus infection model (110).

Interestingly, LNFPIII and Lewis X glycoconjugates can also inhibit TLR signaling in innate immune cells through possible involvement of c-type lectins (132). LNFPIII is a very well-studied HMO that has been linked to many different effects including hepatosteatosis and insulin resistance (136), autoimmunity (137), and transplantation (138). Especially in the case of insulin resistance and autoimmunity, HMOs have been shown to elicit a protective effect in a murine model of Type 1 Diabetes (T1D) (139). The paper shows that supplementation of HMOs can alter microbiota composition and SCFA production in a NOD-mouse model that can prevent spontaneous progression to diabetes. The protective effect of SCFA-producing diets on T1D has been documented beforehand (140, 141). On the other hand, by increasing barrier integrity HMOs may also reduce gut permeability, which has been argued to contribute to the onset of T1D (142).

### Indirect effects of HMOs on intestinal epithelium and immune function via SCFA

Another important role of HMOs is establishing and maintaining the intestinal microbiota. Breastfed infants have higher numbers of beneficial bifidobacteria and lactobaccilli than bottle fed infants. This is the result of preferential fermentation of HMOs by the microbiota by bifidobacterial and lactobacilli (143–145). Upon fermentation of HMOs these bacteria produce, in addition to lactic acid, the short chain fatty acids (SCFA) butyrate, actetate, and propionate. These SCFA improve intestinal barrier function (146) lower the pH in the colon, and have well established anti-inflammatory properties (147).

The notion that composition and metabolic activity of the intestinal microbiota affects the development of allergies has become clearer over the last years (148–151). Exactly how the microbiota composition influences allergy development is not clear at this point, but data from animal models strongly suggest a protective role for SCFA (152–155). A similar role was shown for the SCFA receptors GPR43 in asthma, arthritis, and colitis models (156), and for GPR41 in allergic airway inflammation.

Taken together, these data suggests that HMOs may also have an indirect effect on allergy.

## HMOs and allergy

Several, but not all, studies on the association between breastfeeding and allergy have shown effects on allergic outcomes (157–159). One of the factors that may explain the conflicting findings described above may be the result of differences in breastmilk composition (in relation to milk proteins) (160). None of these studies have correlated their findings with HMO composition.

However, two studies have done just this. In a cohort study of cow's milk allergy (CMA) (9) it was observed that the concentration of 6SL, DSLNT, LNFPI, and LNFPIII was lower in the breast milk of mothers having infants with CMA. After further corrections, only breast milk levels of LNFPIII associated reversely and significantly with development of CMA. In the same study it was observed that FUT2 status of mothers correlated with a delayed onset of CMA while CMA infants born to non-secretor mothers (FUT2 negative) were prone to acute CMA (IgE-mediated). FUT2 status seemed to play a role also in IgE-mediated eczema developed in infants born with C-section (70). In a study of 266 infants followed for 5 years, they observed that infants born to secretor mothers had lower incidence of IgE-mediated eczema. That effect was evident at 2 years but not at 5 years of age though. 2′-FL (one of the main HMOs produced by secretor mothers) was shown to have a significant association with any allergic disease, acute or delayed in infants born with C-section in the same study. 2′-FL and 6′SL had also a beneficial effect in a mouse model of OVA-induced allergy (161). Both HMOs could increase numbers of IL10 producing Treg cells and alleviate allergic symptoms but through different mechanisms.

## Conclusions

**Figure 2:**
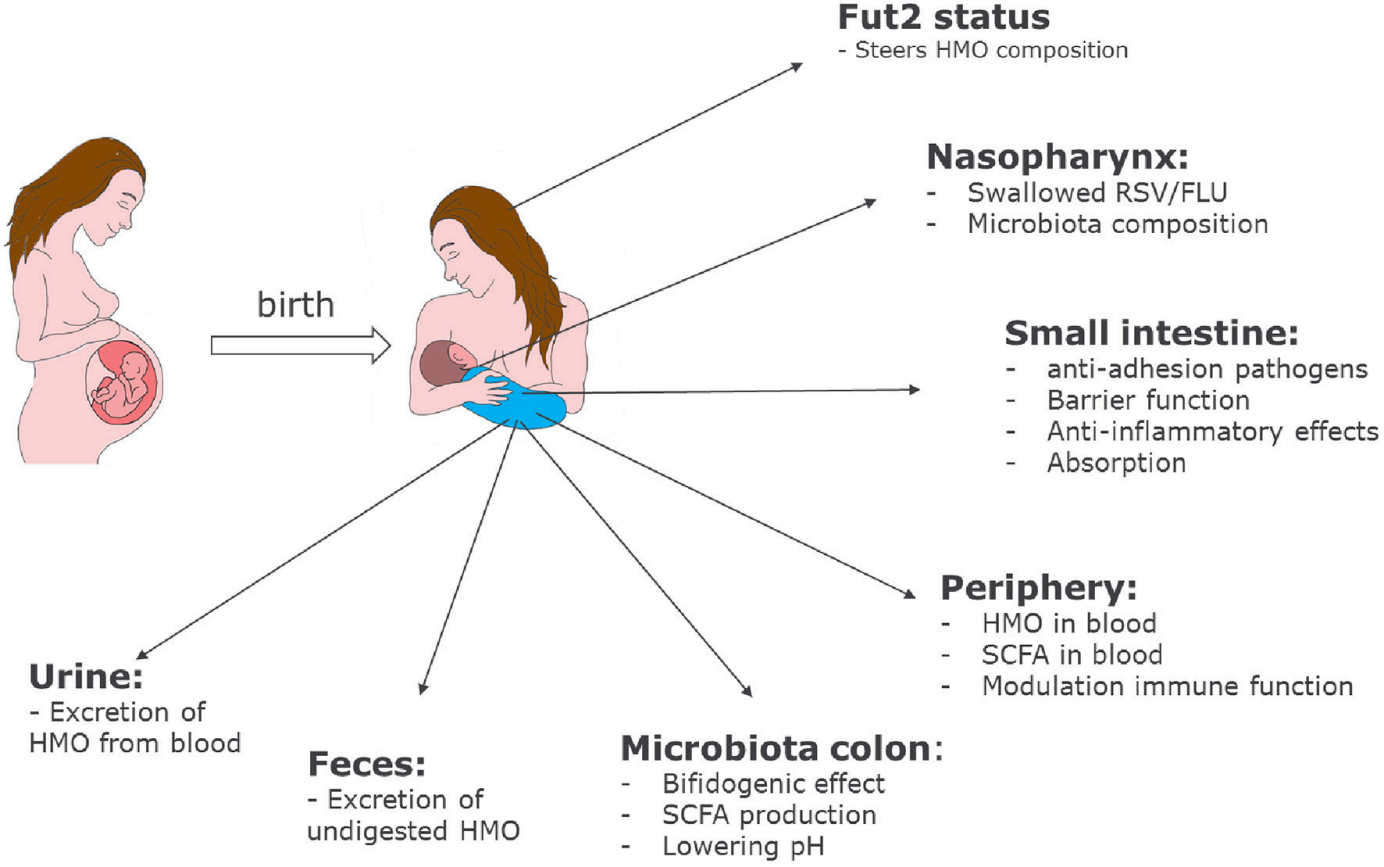
Role of HMOs in developmental physiology of infants: Depending on the genetic background of the mother, HMO composition may differ. Depending on the HMO composition in breast milk, benefits of HMOs span a broad range from shaping the infant microbiome to preventing infections and having systemic effects in the infant after absorption in the intestine.

HMOs contribute to the development of the microbiota and the immune system of newborn infants. The mechanisms by which HMOs contribute have become clearer over the past few years, and our current knowledge is summarized in Figure 2.

However, despite many *in vitro-* and animal experiments, HMOs have not been tested extensively in placebo controlled infant studies. It is clear that several HMOs will be introduced in the near future into infant nutrition to supplement or replace non-human prebiotics like galactooligosaccharides and/or fructooligosaccharides. Prebiotics have been added to infant nutrition in the early 2000's as non-digestible oligosaccharides in an attempt to mimic some of the function of HMOs. With these prebiotics a large number of studies have shown effects on intestinal infection, respiratory infection and allergy (162–167). As the selection of prebiotics is based on functional similarities with HMOs, and extrapolating from *in vitro* and animal experiments with HMOs, it is to be expected that inclusion of HMOs to infant formula will have additional benefits to infant health, and may supplement the functionality of the prebiotics that are already used. Still more research is needed to clarify whether HMOs may also have a therapeutic rather than a protective effect in human immune disorders. Our emerging evidence for the beneficial effects of HMOs once again provide a powerful rationale to encourage women to breastfeed their infants to provide the full scope of benefits that stem from a diverse composition of HMOs that is provided through mother's milk and could potentially be personalized to match the genetic context and environmental exposures of the mother-infant dyad.

## Author contributions

All authors listed have made a substantial, direct and intellectual contribution to the work, and approved it for publication.

### Conflict of interest statement

VT and RvN are employees of FrieslandCampina. The remaining author declares that the research was conducted in the absence of any commercial or financial relationships that could be construed as a potential conflict of interest.
